# Editorial: New advances in understanding the regulation of appetite in insects

**DOI:** 10.3389/finsc.2024.1394092

**Published:** 2024-03-13

**Authors:** Christopher Mayack, Marion Le Gall, Kate Ihle

**Affiliations:** ^1^ Molecular Biology, Genetics, and Bioengineering, Faculty of Engineering and Natural Sciences, Sabancı University, Istanbul, Türkiye; ^2^ US Department of Agriculture, Invasive Species and Pollinator Health Research Unit (ISPHRU), Western Regional Research Center (WRRC) in the Pacific West Area (PWA), Davis, CA, United States; ^3^ M3 Agriculture and Technologies, Tempe, AZ, United States; ^4^ Honey Bee Breeding, Genetics, and Physiology Laboratory, United States Department of Agriculture - Agricultural Research Services (USDA-ARS), Baton Rouge, LA, United States

**Keywords:** nutrients & energy, energetic homeostasis, nutrient intake, foraging preferences, feeding behavior

Appetite regulation is a key process for the maintenance of energetic homeostasis and securing nutrients necessary to carry out life functions, a challenge faced by all organisms (1, 2). Insects are the largest and most diverse group of animals on Earth. They can be fungivores, herbivores (including pollinators), parasitoids, predators, or saprophytes in a huge range of terrestrial habitats spanning all seven continents (3). Despite this variation in habitat and feeding habits, maintaining energetic and nutritional homeostasis at the organismal and physiological level, remains a conserved evolutionary problem (4–7), so how do insects achieve optimal energy intake and nutritional balance with very little food storage buffering capacity, due to their small size? Similarly to vertebrates, insects tackle this challenge through pre- and post-ingestive mechanisms (8). However, given their small size and high levels of activity, do insects have more efficient feedback mechanisms for maintaining energetic homeostasis and optimal nutritional profiles in comparison to vertebrates?

Insects forage in complex and heterogeneous nutritional landscapes, while balancing specific nutrient blend to maximize growth and fitness (9). It has been demonstrated that many insect species have evolved the ability to select between different foods to balance their nutrient intake (10). However, the capacity to detect amino acids has never been shown for the house cricket (*Acheta domesticus*), a common model organism for both research and teaching. In this Research Topic, Tierney et al. established for the first time that house crickets can discriminate essential amino acids (EAA) from sucrose and are capable of increasing amino acid consumption to compensate for dietary unbalance. They showed that EAA/sucrose regulation is linked to reproduction as preference for EAA was found to be stronger for female crickets (juveniles and virgins) over males. On the other hand, mated females preferred sucrose prior to and after egg laying.

Unlike predicted by the nitrogen limitation theory, migratory herbivorous insects, must fuel their energy-demanding flight activities *via* an increase in carbohydrate consumption, as opposed to consuming protein (11). Evidence from the literature suggests that mechanical and chemical defenses of plants, can potentially limit carbohydrate intake for herbivores (12). Here, Brosemann et al. demonstrated that migratory Australian plague locust (*Chortoicetes terminifera*) does in fact prefer plants with a lower protein-carbohydrate ratio (unfertilized plants), adding to the growing body of evidence that migratory insects may be carbohydrate limited. Unlike other studies, they did not find that mechanically grinding plant cell walls improved insect performance, perhaps because the plants they used had softer tissues or did not contain as many carbohydrates as the other plant species tested.

The timing of nutrient regulation can be critical as well. Typically, an insect must ingest an optimal balance of multiple nutrients or face fitness consequences for failing to do so. However, little is known about the timeframe in which insects must secure an optimal nutrient intake before a fitness cost occurs. In this study, Deans and Hutchison, used the spotted-wing Drosophila (*Drosophila suzukii*), an animal impacted not only by phenological change in plant nutrients, but also microbial colonization, to show that feeding intervals and timing of resource availability have strong impacts on foraging, nutrient regulation, and fitness. They found that flies fed at shorter intervals (i.e. diets were changed more frequently) ate more and lived longer. More specifically, the lifespan data show that being able to regulate within a 24-hr period is preferable to longer periods. The flies in the longer-interval treatments were able to regulate their macronutrient intake as precisely as those in the shorter-interval treatments. This suggests that the physiological mechanisms that underlie nutrient regulation operate effectively over short and long timeframes. Understanding these mechanisms will be important for predicting insect responses to changes in resource availability, whether this is from natural causes or anthropogenically induced ones, such as global climate change.

Hunger, energetic demand, and appetite levels, the desire for food intake, tend to be correlated with one another, but how the two crosstalk and regulate one another, is not well known (13). Insects, in contrast to vertebrates, tend to have trehalose as the primary sugar found in the hemolymph (14), which could serve as an alternative monitor of the energetic state in comparison to glucose levels in the blood found with vertebrates (15). Ghanem et al. found that hemolymph trehalose levels aid in regulating appetite levels in forager bees *via* octopamine and tyramine levels in the brain. Surprisingly, this regulatory pathway appears to be functioning independently of the glucose-insulin signaling pathway. The findings suggest that forager honey bees may have evolved a more direct, precise, and rapid regulatory pathway for energy intake in comparison to vertebrates. Whether this newly recognized regulatory appetite pathway found in forager honey bees can be generalized to other insects, which also undergo energy-demanding activities, such as flight, remains to be investigated.

With this Research Topic, we gain exciting insights into how insects manage to account for their nutritional and energetic needs in complex and heterogenous food landscapes. This Research Topic features a new insect species capable of sensing EAA, the concept of carbohydrate limitation for migratory species of herbivorous insects, the importance of time in nutrient regulation, and molecular insights into the regulation of appetite. Taken together, these recent findings reveal the impressive strategies insects adopt to secure optimal nutrition and energy levels in a dynamic environment.

## Author contributions

CM: Writing – original draft, Writing – review & editing. ML: Writing – original draft, Writing – review & editing. KI: Writing – original draft, Writing – review & editing.

## References

[1] Simpson SJ Bernays EA. The Regulation of Feeding: Locusts and Blowflies are not so Different from Mammals. Appetite. (1983) 4:313–46. doi: 10.1016/S0195-6663(83)80024-5 6670861

[2] Erlanson-Albertsson C. Appetite regulation - does it exist? Näringsforskning. (2000) 44:12–4. doi: 10.3402/fnr.v44i0.1780

[3] Klowden M. Physiological Systems in Insects. New York: Academic Press (2013). doi: 10.1016/B978-0-12-415819-1.00006-4

[4] Raubenheimer D Simpson SJ Mayntz D. Nutrition, ecology and nutritional ecology: toward an integrated framework. Funct Ecol. (2009) 23:4–16. doi: 10.1111/j.1365-2435.2009.01522.x

[5] Vézina F Salvante KG. Behavioral and physiological flexibility are used by birds to manage energy and support investment in the early stages of reproduction. Curr Zoology. (2010) 56:767–92. doi: 10.1093/czoolo/56.6.767

[6] Zhao ZJ Chi QS Cao J Han YD. The energy budget, thermogenic capacity and behavior in Swiss mice exposed to a consecutive decrease in temperatures. J Exp Biol. (2010) 213:3988–97. doi: 10.1242/jeb.046821 21075940

[7] Mayack C Naug D. Individual energetic state can prevail over social regulation of foraging in honeybees. Behav Ecol Sociobiology. (2013) 67:929–36. doi: 10.1007/s00265-013-1517-6

[8] Schneider JE Wise JD Benton NA Brozek JM Keen-Rhinehart E. When do we eat? Ingestive behavior, survival, and reproductive success. Hormones Behav. (2013) 64:702–28. doi: 10.1016/j.yhbeh.2013.07.005 23911282

[9] Raubenheimer D Simpson SJ. The geometry of compensatory feeding in the locust. Anim Behav. (1993) 45:953–64. doi: 10.1006/anbe.1993.1114

[10] Waldbauer GP Friedman S. Self-selection of optimal diets by insects. Annu Rev Entomology. (1991) 36:43–63. doi: 10.1146/annurev.en.36.010191.000355

[11] Talal S Cease AJ Youngblood JP Farington R Trumper EV Medina HE. Plant carbohydrate content limits performance and lipid accumulation of an outbreaking herbivore. Proc R Soc B: Biol Sci. (2020) 287:20202500. doi: 10.1098/rspb.2020.2500 PMC7739925 33259763

[12] War AR Paulraj MG Ahmad T Buhroo AA Hussain B Ignacimuthu S. Mechanisms of plant defense against insect herbivores. Plant Signal Behav. (2012) 7:1306–20. doi: 10.4161/psb.21663 PMC3493419 22895106

[13] Austin J Marks D. Hormonal regulators of appetite. Int J Pediatr Endocrinol. (2009) 2009:141753. doi: 10.1186/1687-9856-2009-141753 19946401 PMC2777281

[14] Thompson SN. Trehalose - The insect ‘blood’ sugar. Adv Insect Physiol. (2003) 31:205–85. doi: 10.1016/S0065-2806(03)31004-5

[15] Akülkü İ Ghanem S Filiztekin E Suwannapong G Mayack C. Age-dependent honey bee appetite regulation is mediated by trehalose and octopamine baseline levels. Insects. (2021) 12:1–12. doi: 10.3390/insects12100863 PMC8539172 34680632

